# Psychotherapeutic intervention for treatment of psychotic symptoms in patients with paranoid development. About a case

**DOI:** 10.1192/j.eurpsy.2022.2042

**Published:** 2022-09-01

**Authors:** C. Martín Villarroel, L. Carpio García, M. Sánchez Revuelta, J. Matsuura, G. Belmonte García, L. Santolaya López, E.F. Benavides Rivero

**Affiliations:** Complejo Hospitalario Universitario de Toledo, Psiquiatría, Toledo, Spain

**Keywords:** Trauma, mentalization-based therapy, Psychotherapy, psychotic symptoms

## Abstract

**Introduction:**

Psychotic symptoms are not exclusive to schizophrenia, they can be due to paranoid development and can be treated differently.

**Objectives:**

The objective of this paper is to study, from the following case, the effect of psychotherapeutic treatment in patients with paranoid development.

**Methods:**

A bibliographic search was performed from different database (Pubmed, TripDatabase) about psychological intervention for the improvement of paranoid symptoms. 20-year-old man, born into a family with marital problems, without difficulties in psychomotor development, socialization or academic performance, who began with behavioral alterations from the age of 5 that he had begun to suffer abuse from his father, showing aggressiveness towards other children and progressively worsening over the years: consuming cannabis, isolating himself, listening to protective voices and distrusting of people, to whom he responded aggressively believing that they wanted to harm him.

**Results:**

Initially, he was treated with antipsychotics that were later suspended when acute psychotic symptoms were ruled out, diagnosing a paranoid development secondary to trauma, for which he had felt fear and defenselessness, and had learned to be alert and respond aggressively to everything he considered threatening, showing anger that he did not know how to express. During therapy, abstinence to drugs was worked on, therapeutic link, mentalization-based therapy, emotions, narrative techniques, trauma and systemic family therapy.

**Conclusions:**

To conclude, we need to pay attention to development of pathologies like this so as not to rush with antipsychotics, when it may be due to a development secondary to trauma that needs to be treated psychotherapeutically.

**Disclosure:**

No significant relationships.

